# Renal sulfate reabsorption in healthy individuals and renal transplant recipients

**DOI:** 10.14814/phy2.13670

**Published:** 2018-04-19

**Authors:** Adrian Post, Isidor Minović, Else van den Berg, Manfred L. Eggersdorfer, Gerjan J. Navis, Johanna M. Geleijnse, Reinold O. B. Gans, Harry van Goor, Joachim Struck, Casper F. M. Franssen, Ido P. Kema, Stephan J. L. Bakker

**Affiliations:** ^1^ Department of Internal Medicine University Medical Center Groningen University of Groningen Groningen The Netherlands; ^2^ Top Institute Food and Nutrition Wageningen The Netherlands; ^3^ Department of Laboratory Medicine University Medical Center Groningen University of Groningen Groningen The Netherlands; ^4^ DSM Nutritional Products Kaiseraugst Switzerland; ^5^ Division of Human Nutrition Wageningen University Wageningen The Netherlands; ^6^ Department of Pathology University Medical Center Groningen University of Groningen Groningen The Netherlands; ^7^ SphingoTec GmbH Hohen Neuendorf Germany; ^8^ Transplant Lines Food and Nutrition Biobank and Cohort Study University Medical Center Groningen University of Groningen Groningen The Netherlands

**Keywords:** Kidney donation, renal sulfate handling, renal transplant recipients, sulfate reabsorption

## Abstract

Inorganic sulfate is essential for normal cellular function and its homeostasis is primarily regulated in the kidneys. However, little is known about renal sulfate handling in humans and particularly in populations with impaired kidney function such as renal transplant recipients (RTR). Hence, we aimed to assess sulfate reabsorption in kidney donors and RTR. Plasma and urinary sulfate were determined in 671 RTR and in 251 kidney donors. Tubular sulfate reabsorption (TSR) was defined as filtered load minus sulfate excretion and fractional sulfate reabsorption (FSR) was defined as 1‐fractional excretion. Linear regression analyses were employed to explore associations of FSR with baseline parameters and to identify the determinants of FSR in RTR. Compared to kidney donors, RTR had significantly lower TSR (15.2 [11.2–19.5] vs. 20.3 [16.7–26.3] *μ*mol/min), and lower FSR (0.56 [0.48–0.64] vs. 0.64 [0.57–0.69]) (all *P* < 0.001). Kidney donation reduced both TSR and FSR by circa 50% and 25% respectively (both *P* < 0.001). In RTR and donors, both TSR and FSR associated positively with renal function. In RTR, FSR was independently associated with urinary thiosulfate (*β* = −0.18; *P* = 0.002), growth hormone (*β* = 0.12; *P* = 0.007), the intakes of alcohol (*β* = −0.14; *P* = 0.002), methionine (*β* = −0.34; *P* < 0.001), cysteine (*β* = −0.41; *P* < 0.001), and vitamin D (*β* = −0.14; *P* = 0.009). In conclusion, TSR and FSR are lower in RTR compared to kidney donors and both associated with renal function. Additionally, FSR is determined by various dietary and metabolic factors. Future research should determine the mechanisms behind sulfate handling in humans and the prognostic value of renal sulfate reabsorption in RTR.

## Introduction

Inorganic sulfate is the fourth most abundant anion in human plasma, and its concentration is primarily determined by renal excretion and reabsorption (Goudsmit et al. 1939; Hierholzer et al. 1960). The indispensable role of sulfate in normal cell function is reflected by its involvement in a wide variety of physiological processes, such as biosynthesis of proteoglycans, activation of endogenous compounds, for example, heparin, gastrin, and cholecystokinin, and metabolism and detoxification of various endogenous substances and xenobiotics (Falany 1997; Cole and Evrovski 2000; Lee et al. 2000; Dawson et al. 2003, 2015; Markovich and Aronson 2007). While the key regulatory processes of renal sulfate handling have been extensively studied in animals and cell lines (Renfro and Dickman 1980; Leyh et al. 1992; Cherest et al. 1997; Smith et al. 2000), very little is known about these mechanisms in humans. In 1960, a study that measured sulfate reabsorption in 16 healthy adults before and after infusion of sodium sulfate (Becker et al. 1960), showed that tubular sulfate reabsorption was saturable and varied markedly between individuals. However, the effect of renal function on renal sulfate reabsorption remains, to date, unknown. Additionally, factors that have been shown to influence tubular sulfate reabsorption in animals and cell lines, for example, growth hormones, thyroid hormone, and nonhormonal factors, for example, dietary sulfate, vitamin, and NSAID intake, have not been assessed in humans (Sabry et al. 1965; Frick and Durasin 1986; Neiberger 1991; Tenenhouse et al. 1991; Fernandes et al. 1997; Sagawa et al. 1998a,b, 1999a,b; Markovich et al. 1999).

To investigate sulfate reabsorption in humans, we measured sulfate reabsorption in kidney donors, before and after donation and in a well‐characterized cohort with a large variation in renal function, that is, renal transplant recipients (RTR), allowing us to (1) assess and compare sulfate reabsorption in kidney donors before donations and RTR, (2) investigate the influence of kidney donation on sulfate reabsorption in kidney donors, (3) determine the influence of renal function on sulfate reabsorption in kidney donors and RTR, and (4) identify the determinants of sulfate reabsorption in RTR.

## Materials and Methods

### Study population

This cross‐sectional study was based on a previously described, well‐characterized set of 707 RTR (van den Berg et al. 2012, 2014.) In brief, this cohort included RTR (aged ≥ 18 years) who visited the outpatient clinic of the University Medical Center Groningen (UMCG), Groningen, the Netherlands, between November 2008 and June 2011 and who had a graft that had been functioning for at least 1 year with no history of alcohol and/or drug addiction. We excluded subjects with missing data on tubular sulfate reabsorption (TSR) and fractional sulfate reabsorption (FSR), that is, 36 cases, from the statistical analyses, which resulted in 671 cases eligible for analyses. As a control group, we included 251 healthy kidney donors of whom biomaterial was collected before and after kidney donation, with 3 months between collections. The study protocol was approved by the UMCG institutional review board (METc 2008/186) and adhered to the Declarations of Helsinki.

### Data collection and measurements

Information on dietary intake was obtained from a validated semi‐quantitative food frequency questionnaire, which was developed at Wageningen University to assess nutrient intake (Eisenga et al. 2016). Because not all participants completed or returned the FFQ, only 643 of the 671 RTR had data available on dietary intake derived from the FFQ, (whereas 671 RTR had plasma sulfate concentrations, TSR and FSR available). The FFQ inquired about intake of 177 food items during the last month, taking seasonal variations into account. For each item, the frequency was recorded in times per day, week, or month. The number of servings was expressed in natural units (e.g., slice of bread or apple) or household measures (e.g., cup or spoon). The questionnaire was self‐administered and filled out at home. All FFQs were checked for completeness by a trained researcher, and inconsistent answers were verified with the patients. Validation of the FFQ in RTR was assessed as previously reported (Oste et al. 2017). Dietary data were converted into daily nutrient intake using the Dutch Food Composition Table of 2006 (National Institute for Public Health and the Environment, 2013.) Medication use was determined using patients’ medical records.

Participants were asked to collect a 24‐h urine sample on the day prior to visiting the outpatient clinic. Urine was collected under oil, and chlorhexidine was added as an antiseptic agent. Urinary protein concentration was determined by means of the Biuret reaction (MEGA AU 510; Merck Diagnostica, Darmstadt, Germany). Proteinuria was defined as urinary protein excretion ≥0.5 g/24 h.

Upon completion of the 24‐h urine collection, fasting blood samples were obtained the following morning, and venous blood samples were analyzed spectrophotometrically immediately thereafter. Plasma sulfate and urinary sulfate were measured by means of a validated ion‐exchange chromatography assay with conductivity detection (Metrohm, Herisau, Switzerland). Growth hormone was assessed with a high sensitivity chemiluminescence sandwich immunoassay (SphingoTec GmbH, Borgsdorf, Germany), as described elsewhere (Hallengren et al. 2014). Other laboratory measurements were performed with automated and validated routine methods (Roche Diagnostics, Basel, Switzerland). Diabetes (mellitus) was diagnosed according to American Diabetes Association criteria as fasting plasma glucose concentration of at least 7.0 mmol/L or use of antidiabetic medication. (Abbasi et al. 2012)

TSR was calculated to align results of our current analyses with existing literature in the field of sulfate reabsorption dating from 1960 (Becker et al. 1960), where data for TSR were presented. TSR was expressed as absolute tubular sulfate reabsorption and was defined as the filtered load minus the sulfate excretion, calculated as:

$$\text{TSR}\;\left(\mu \text{mol/min}\right)={P_{{\mathrm{SO}}_{4}}}^{*}\text{GFR}-{U_{{\mathrm{SO}}_{4}}}^{*}V$$

where U_SO4_ and P_SO4_ represent urinary and plasma concentrations of sulfate in µmol/mL; GFR represents glomerular filtration rate in mL/min; and V represents urine flow in mL/min.

Measures of tubular reabsorption, including TSR, typically depend strongly on renal function. Therefore, it is currently more common to correct for renal function, using the fractional reabsorption, that is, FSR in case of sulfate. FSR was defined as the TSR divided by the filtered load and was calculated as follows:

$$\text{FSR}\;\left(\%\right)=\text{TSR}/{\left({\text{GFR}}^{}*P_{{\mathrm{SO}}_{4}}\right)}^{*}100$$

When GFR is approximated by creatinine clearance, FSR mathematically corresponds to 1 minus the fractional sulfate excretion, calculated as follows:

$$\text{FSR}={\left[1-\left({U_{{\mathrm{SO}}_{4}}}^{*}P_{\mathrm{creat}}\right)/\left({P_{{\mathrm{SO}}_{4}}}^{*}U_{\mathrm{creat}}\right)\right]}^{*}100\%$$

where P_creat_ and U_creat_ represents urinary and plasma concentrations of creatinine in µmol/mL. In RTR, TSR and FSR were assessed using GFR based on creatinine clearance, since no ^125^I‐Iodothalamate data were available for RTR. In kidney donors, TSR and FSR were assessed twice, that is, using GFR based on creatinine clearance for comparison with RTR, and using GFR assessed by the urinary clearance of ^125^I‐Iothalamate method for comparing pre‐ and post‐donation parameters. For assessment of the associations with renal function, TSR and FSR were calculated using creatinine clearance in RTR and ^125^I‐Iothalamate in kidney donors.

### Statistical analyses

Data analysis was performed using SPSS 22.0 for Windows (SPSS Inc., Chicago, IL), GraphPad Prism version 5.01 for Windows (GraphPad Software, San Diego, CA), and R version 3.2.3 (The R‐Foundation for Statistical Computing, Vienna, Austria). In R, generalized additive models of the mgcv package were used to model the associations of plasma sulfate with TSR and FSR. Model effect and nonlinearity were tested by two‐sided Wald tests. *P*_nonlinearity_ was calculated by comparing restricted cubic spline terms to a linear model.

Data are presented as mean ± standard deviation (SD) for normally distributed data, as median [interquartile range (IQR)] for non‐normally distributed data, and as number (percentage) for nominal data. A two‐sided *P* < 0.05 was considered to indicate statistical significance.

Differences between RTR and kidney donors in plasma sulfate, TSR and FSR were tested using linear regression analysis. This type of analysis was also employed to investigate cross‐sectional associations of FSR with baseline variables (*P*_trend_). Mann–Whitney *U* test and independent *t*‐test were used to assess the differences in sulfate parameters before and after donation in kidney donors.

Associations of TSR and FSR with renal function parameters were tested using linear regression analysis. Additional adjustments were made for age and gender. To check for interaction between age and renal function, and gender and renal function, interaction terms were calculated and checked for significance in a linear regression model with TSR and FSR, separately.

Since TSR depends more on renal function than FSR, FSR was used for determinant analysis. Determinants of FSR were identified in a multivariable regression model, in which variables that were suggested to affect FSR in animals and cell lines, were included. These variables included plasma sulfate, thiosulfate excretion, intake of alcohol, water, bread, fruit, methionine, cysteine, vitamin D, use of NSAID, serum potassium, venous pH, venous HCO_3_^−^, net acid excretion, thyroid hormones and growth hormone. In the multivariable models, adjustments were made for common confounders such as age, gender, smoking, BMI, proteinuria, total energy intake, and primary renal disease.

To check for interaction between age and growth hormone, and gender and growth hormone, interaction terms were calculated and checked for significance in a linear regression model with TSR and FSR, separately.

## Results

### Sulfate reabsorption in RTR and kidney donors before donation

**Figure 1:**
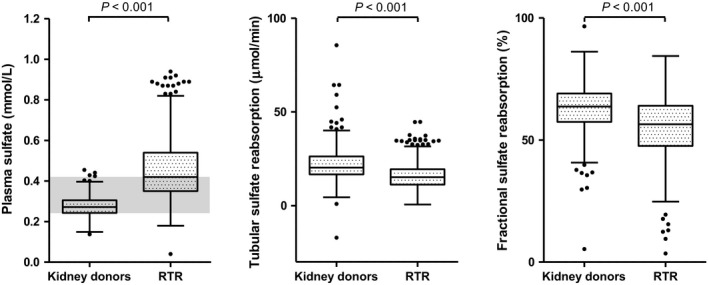
Plasma sulfate concentrations, tubular sulfate reabsorption and fractional sulfate reabsorption in 251 kidney donors before kidney donation and in 671 renal transplant recipients. RTR, renal transplant recipients.

Mean age of RTR at inclusion (5.3 (1.8–12.1) years after transplantation) was 53 ± 13 years and 57% were male, compared to 54 ± 11 years and 47% males for kidney donors, respectively. Differences in plasma sulfate, TSR and FSR between kidney donors and RTR are displayed Figure 1. Median plasma sulfate [interquartile range (IQR)] in kidney donors was 0.28 [0.24–0.31] mmol/L, compared to 0.43 [0.35–0.54] mmol/L in RTR (*P* < 0.001). Kidney donors had higher TSR than RTR, 20.3 [16.7–26.3] µmol/min versus 15.2 [11.2–19.5], (*P* < 0.001), respectively. Furthermore, kidney donors also had higher FSR than RTR, 0.64 [0.57–0.69] versus 0.56 [0.48–0.64] (*P* < 0.001), respectively.

### Sulfate reabsorption before and after kidney donation in kidney donors

**Figure 2:**
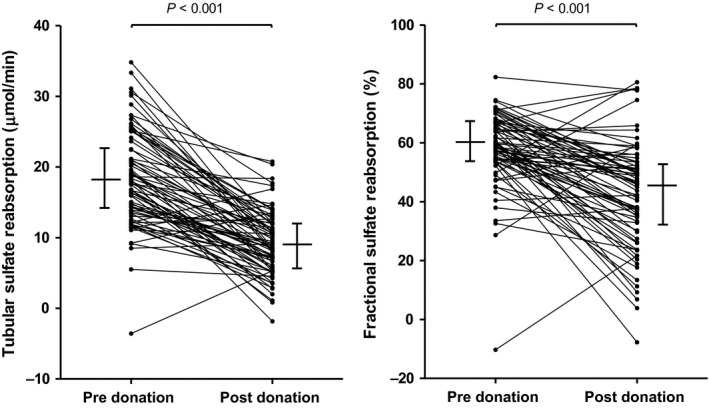
Tubular and fractional sulfate reabsorption before and after kidney donation in 251 kidney donors. Tubular and fractional sulfate reabsorption were calculated using the urinary clearance of 125I‐Iothalamate method as GFR.

Sulfate parameters in kidney donors, before and after donation, are shown in Table 1. Kidney donation caused a reduction in renal function (113 ± 22 to 74 ± 13; *P* < 0.001) and sulfate excretion in kidney donors (17.7 [14.4–20.8] vs. 17.2 [12.2–22.0]; *P* < 0.001). Furthermore, after kidney donation, plasma sulfate concentration increased significantly (0.27 [0.24–0.31] vs. 0.29 [0.26–0.32]; *P* < 0.001), while TSR (18.8 ± 6.3 vs. 9.3 ± 4.5; *P* < 0.001) and FSR (60 [54–67] vs. 45 [32–53]; *P* < 0.001) were reduced, Figure 2.

**Table 1:** Sulfate parameters in kidney donors before and after kidney donation

	Pre‐donation	Post‐donation	*P* for difference
Plasma sulfate (mmol/L)	0.27 [0.24–0.31]	0.29 [0.26–0.32]	<0.001
Sulfate excretion (mmol/24 h)	17.7 [14.4–20.8]	17.2 [12.2–22.0]	<0.001
GFR (mL/min/1.73 m^2^)	113 ± 22	74 ± 13	<0.001
Sulfate clearance (mL/min)	43.9 [35.9–53.4]	39.3 [31.2–50.2]	<0.001
Fractional sulfate excretion (%)	40 [33–46]	55 [47–68]	<0.001
Filtered sulfate load (*μ*mol/min)	30.3 [26.3–36.0]	22.1 [189–25.0]	<0.001
Tubular sulfate reabsorption (*μ*mol/min)	18.8 ± 6.3	9.3 ± 4.5	<0.001
Fractional sulfate reabsorption (%)	60 [54–67]	45 [32–53]	<0.001
Tubular potassium reabsorption (*μ*mol/min)	361 [314–437]	231 [201–272]	<0.001
Fractional potassium reabsorption (%)	0.86 [0.83–0.89]	0.80 [0.76–0.84]	<0.001
Tubular sodium reabsorption (mmol/min)	15.7 [13.8–18.0]	10.2 [9.0–11.6]	<0.001
Fractional sodium reabsorption (%)	0.99 [0.99–0.99]	0.99 [0.99–0.99]	<0.001

*P* value for difference was tested by Wilcoxon Signed‐Rank test or paired sample *t*‐test. Tubular and fractional sulfate reabsorption were calculated using the urinary clearance of 125I‐iothalamate method as the measured GFR.

### Sulfate reabsorption and renal function in kidney donors and RTR

In kidney donors before donation, mGFR was 111 [98–128] mL/min/1.73 m^2^_._ TSR (*β* = 0.50; *P* < 0.001), but not FSR (*β* = 0.11; *P* = 0.099) was associated with mGFR in the crude model. After adjustment for age and gender, both TSR (*β* = 0.65; *P* < 0.001) and FSR (*β* = 0.25; *P* = 0.003) associated with mGFR. No significant interaction between gender and mGFR, nor age and mGFR was found, in the associations with TSR and FSR respectively.

In RTR, the creatinine clearance was 64 [46–83] mL/min. Both TSR (*β* = 0.54; *P* < 0.004) and FSR (*β* = 0.09; *P* = 0.019) was associated with creatinine clearance in the crude model. After adjustment for age and gender, TSR (*β* = 0.57; *P* < 0.001) and FSR (*β* = 0.13; *P* = 0.001) this association remained significant. No significant interaction between gender and creatinine clearance, nor age and creatinine clearance was found, in the associations with TSR and FSR, respectively.

### Baseline characteristics in RTR

**Figure 3:**
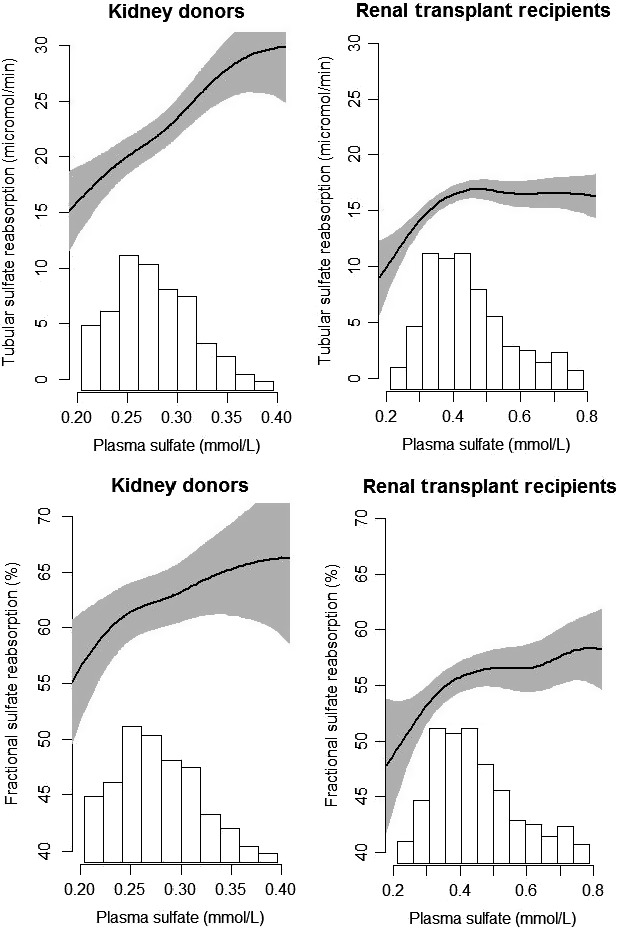
Associations between plasma sulfate, tubular sulfate reabsorption, and fractional sulfate reabsorption in 251 kidney donors before donation and in 671 renal transplant recipients. Tubular and fractional sulfate reabsorption were calculated with creatinine clearance. Tubular sulfate reabsorption in kidney donors *P*_effect_ < 0.001; *P*_nonlineariry_ = 0.20; tubular sulfate reabsorption in RTR *P*_effect_ < 0.001; *P*_nonlineariry_ < 0.001; fractional sulfate reabsorption in kidney donors *P*_effect_ = 0.05; *P*_nonlineariry_ = 0.13; fractional sulfate reabsorption in RTR *P*_effect_ = 0.01; *P*_nonlineariry_ = 0.16.

Baseline characteristics of the total RTR cohort and according to tertiles of FSR are shown in Table 2. FSR was positively associated with age, BMI, deceased kidney donor (verses living donor), hs‐CRP, NT‐proBNP, use of antihypertensives and diuretics, and growth hormone (all *P* < 0.05). Inverse associations were observed between FSR and sulfate excretion, thiosulfate excretion, male gender, intake of alcohol, water, bread, fruit, animal protein, the sulfur‐containing amino acids methionine and cysteine, vitamin D, and energy, as well as with renovascular disease, serum creatinine and net acid excretion (all *P* < 0.05). Additionally, we observed a positive nonlinear association between plasma sulfate and TSR and FSR, as represented by Figure 3.

**Table 2:** Baseline characteristics of renal transplant recipients presented according to gender‐stratified tertiles of fractional sulfate reabsorption

	Total cohort (*N* = 671)	Tertiles of FSR			*P*_trend_
		I 0.04–0.54 (*N* = 222)	II 0.49–0.76 (*N* = 225)	III 0.61–0.84 (*N* = 224)	
FSR, %	0.56 ± 0.12	0.42 ± 0.09	0.57 ± 0.04	0.68 ± 0.05	
Urinary sulfate, mmol/24 h	17.6 ± 6.4	20.8 ± 6.4	17.9 ± 5.4	14.0 ± 4.8	<0.001
Urinary thiosulfate, *μ*mol/24 h	7.1 [4.0–12.0]	8.6 [4.4–13.2]	7.9 [4.3–13.3]	5.9 [3.5–9.3]	<0.001
Demographics					
Age, years	53 ± 13	52 ± 13	53 ± 13	54 ± 13	0.02
Male gender, *n* (%)	384 (57)	128 (57)	128 (57)	128 (57)	
BMI, kg/m^2^	26.0 [23.3–29.3]	25.6 [23.1–28.7]	26.0 [23.6–29.2]	26.5 [23.2–30.1]	0.003
Smokers, *n* (%)					
Never	266 (42)	84 (39)	88 (42)	94 (45)	0.22
Past	285 (45)	102 (48)	101 (49)	82 (39)	0.09
Current	77 (12)	25 (12)	19 (9)	33 (16)	0.45
Dialysis vintage, months	24 [10–47]	24 [9–50]	23 [8–46]	25 [14–50]	0.08
Time since Rtx, years	5.4 [1.8–12.0]	5.0 [1.3–11.3]	6.1 [1.4–12.0]	5.7 [2.9–12.9]	0.06
Deceased donor, *n* (%)	433 (66)	130 (60)	142 (64)	161 (72)	0.003
Creatinine excretion, mmol/24 h	11.3 [9.2–14.0]	11.5 [9.2–14.3]	11.7 [9.6–14.1]	11.0 [8.9–13.9]	0.05
Dietary intake					
Vegetarian, *n* (%)	14 (2)	5 (2)	5 (3)	4 (2)	0.68
Alcohol intake, g/d	3.0 [0.04–11.5]	4.6 [0.1–14.8]	3.4 [0.2–12.5]	1.0 [0.02–8.5]	<0.001
Water intake, g/d	2074 ± 587	2138 ± 599	2093 ± 559	2021 ± 719	0.002
Bread intake, g/d	133 ± 60	137 ± 65	132 ± 53	129 ± 62	<0.001
Fruit, g/d	123 [60–232]	135 [73–239]	124 [66–237]	105 [50–227]	0.04
Vegetables, g/d	91 [51–123]	91 [58–132]	91 [56–1234]	85 [46–116]	0.35
Animal protein, g/d	53 ± 16	53 ± 16	52 ± 14	49 ± 15	0.001
Vegetable protein, g/d	31 ± 10	31 ± 11	30 ± 8	30 ± 11	0.07
Methionine, mg/d	1884 ± 5.9	1923 ± 495	1895 ± 421	1795 ± 496	<0.001
Cysteine, mg/d	1190 ± 307	1220 ± 313	1191 ± 249	1145 ± 312	<0.001
Vitamin D intake, *μ*g/d	4.6 [3.4–5.9]	4.7 [3.4–6.0]	4.7 [3.7–5.8]	4.4 [3.3–5.9]	0.001
Energy intake, kcal/d	2173 ± 656	2174 ± 635	2171 ± 567	2159 ± 687	0.02
Primary renal disease, *n* (%)					
Primary glomeruloslerosis	192 (29)	59 (27)	68 (30)	65 (29)	0.52
Glomerulonephritis	51 (8)	15 (7)	18 (8)	18 (8)	0.54
Tubulointerstitial nephritis	77 (11)	25 (11)	23 (10)	29 (13)	0.26
Polycystic kidney disease	136 (20)	43 (19)	47 (21)	45 (20)	0.81
Hypo‐ or dysplasia	27 (4)	10 (5)	10 (4)	7 (3)	0.07
Renovascular disease	39 (6)	18 (8)	12 (5)	9 (4)	0.01
Glucose homeostasis					
Diabetes, *n* (%)	163 (24)	46 (21)	53 (24)	64 (29)	0.03
Glucose, mmol/L	5.3 [4.8–6.0]	5.3 [4.8–6.0]	5.2 [4.7–5.9]	5.3 [4.8–6.2]	0.85
HbA_1c_, %	6.0 ± 0.8	5.9 ± 0.8	6.0 ± 0.8	6.1 ± 0.9	0.74
Antidiabetics, *n* (%)	105 (16)	30 (14)	34 (15)	41 (16)	0.09
Inflammation					
hs‐CRP, mg/L	1.6 [0.7–4.7]	1.5 [0.6–3.5]	1.7 [0.8–5.3]	1.7 [0.8–5.0]	0.01
Cardiovascular					
NT‐proBNP, ng/L	251 [104–612]	200 [98–615]	235 [95–515]	285 [119–703]	<0.001
SBP, mmHg	136 ± 17	135 ± 18	135 ± 16	137 ± 18	0.40
DBP, mmHg	83 ± 11	83 ± 12	83 ± 10	81 ± 10	0.09
Antihypertensives, *n* (%)	592 (88)	188 (84)	198 (88)	205 (92)	0.04
Diuretic, *n* (%)	245 (36)	71 (32)	97 (43)	103 (46)	<0.001
Potassium sparing	4 (1)	2 (1)	0 (0)	2 (1)	0.88
Loop	138 (21)	42 (19)	47 (21)	49 (22)	0.08
Thiazide	102 (15)	21 (9)	43 (19)	38 (17)	0.11
Total cholesterol, mmol/L	5.1 ± 1.1	5.1 ± 1.1	5.2 ± 1.1	5.1 ± 1.2	0.65
HDL‐cholesterol, mmol/L	1.4 ± 0.5	1.4 ± 0.5	1.4 ± 0.5	1.4 ± 0.5	0.94
LDL‐cholesterol, mmol/L	2.9 [2.3–3.5]	2.9 [2.4–3.5]	3.0 [2.4–3.6]	2.8 [2.3–3.5]	0.54
Triglycerides, mmol/L	1.7 [1.2–2.3]	1.7 [1.2–2.2]	1.7 [1.2–2.3]	1.7 [1.3–2.4]	0.07
Statins, *n* (%)	350 (52)	112 (50)	117 (52)	121 (54)	0.34
NSAID, *n* (%)	127 (19)	40 (18)	45 (20)	42 (19)	0.74
Renal function					
eGFR, mL/min/1.73 m^2^	45 ± 19	44 ± 18	47 ± 19	45 ± 18	0.59
Proteinuria, ≥0.5 g/24 h, *n* (%)	149 (22)	41 (18)	46 (21)	62 (28)	0.12
Metabolic parameters					
Serum potassium, mmol/L	3.98 ± 0.47	3.84 ± 0.46	3.95 ± 0.44	4.03 ± 0.49	0.20
Venous pH	7.4 ± 0.04	7.4 ± 0.04	7.4 ± 0.04	7.4 ± 0.04	0.32
Venous HCO_3_^−^, mmol/L	24.7 ± 3.1	24.5 ± 2.9	24.9 ± 3.1	24.6 ± 3.2	0.83
Net acid excretion, mEq/24 h	61.1 ± 32.6	63.6 ± 34.8	63.0 ± 27.9	56.2 ± 33.8	<0.001
Liver function					
LDH, U/L	198 [170–232]	200 [167–233]	197 [173–235]	197 [170–228]	0.42
Alkaline phosphatase, U/L	68 [54–84]	66 [54–80]	68 [54–85]	69 [54–88]	0.82
Gamma‐GT, U/L	26 [19–41]	26 [19–43]	26 [19–39]	27 [19–42]	0.40
Endocrinology					
Triiodothyronine, pmol/L	4.9 ± 0.8	4.9 ± 0.8	4.9 ± 0.9	4.8 ± 0.7	0.27
Thyroxine, pmol/L	16.1 ± 2.9	16.2 ± 2.6	16.2 ± 3.0	15.8 ± 3.2	0.24
Growth hormone, ng/mL	0.34 [0.10–1.07]	0.27 [0.08–1.01]	0.30 [0.09–0.87]	0.43 [0.12–1.28]	0.002
Immunosuppression					
CNI, *n* (%)	382 (57)	121 (54)	127 (56)	134 (60)	0.05
Proliferation inhibitor, *n* (%)	560 (83)	188 (84)	192 (85)	179 (80)	0.37
Prednisolone, mg/24 h	10.0 [7.5–10.0]	10.0 [7.5–10.0]	10.0 [7.5–10.0]	10.0 [7.5–10.0]	0.36

RTR, renal transplant recipients; RSR, renal sulfate reabsorption, BMI, body mass index; Rtx, renal transplantation; hs‐CRP, high‐sensitive C‐reactive protein; NT‐proBNP, N‐terminal pro‐b‐type natriuretic peptide; SBP, systolic blood pressure; DBP, diastolic blood pressure; HDL, high‐density lipoprotein; LDL, low‐density lipoprotein; NSAID, nonsteroidal anti‐inflammatory drug; eGFR, estimated glomerular filtration rate; LDH, lactate dehydrogenase; gamma‐GT; gamma‐glutamyl transferase; CNI, calcineurin inhibitor.

### Potential determinants of FSR

For relevant hormonal and nonhormonal parameters, their associations with FSR are shown in Table 3. In the multivariable linear regression model, FSR was positively associated with growth hormone (*β* = 0.12; *P* = 0.007) and inversely associated with urinary thiosulfate (*β* = −0.18; *P* = 0.002), net acid excretion (*β* = −0.19; *P* < 0.001), intakes of alcohol (*β* = −0.14; *P* = 0.002), water (*β* = −0.15; *P* = 0.003), bread (*β* = −0.12; *P* = 0.010), vegetable (*β* = −0.12; *P* = 0.005), methionine (*β* = −0.34; *P* < 0.001), cysteine (*β* = −0.41; *P* < 0.001) and vitamin D (*β* = −0.14; *P* = 0.009). No associations were found between FSR and venous pH, serum potassium, thyroid hormones, and NSAID use (*P*≥0.05).

**Table 3:** Regression analyses with potential determinants of fractional sulfate reabsorption in renal transplant recipients

Potential determinants	Univariable models		Multivariable model^a^	
	Stand. beta	*P*‐value	Stand. beta	*P*‐value
Plasma sulfate, mmol/L	0.11	0.011	0.05	0.203
Urinary thiosulfate, *μ*mol/24 h	−0.23	<0.001	−0.18	0.002
Creatinine cleanance (mL/min)	0.10	0.021	0.16	<0.001
Dietary intake				
Alcohol intake, g/d	−0.18	<0.001	−0.14	0.002
Water intake, g/d	−0.13	0.002	−0.15	0.003
Bread intake, g/d	−0.15	<0001	−0.12	0.010
Daily fruit, g/d	−0.08	0.056	−0.12	0.005
Methionine, mg/d	−0.17	<0.001	−0.34	<0.001
Cysteine, mg/d	−0.17	<0.001	−0.41	<0.001
Vitamin D intake, *μ*g/d	−0.12	0.004	−0.14	0.009
Cardiovascular				
NSAID, *n* (%)	0.02	0.628	0.01	0.753
Serum potassium, mmol/L	0.04	0.356	0.04	0.322
Metabolic parameters				
Venous pH	0.01	0.871	0.03	0.534
Venous HCO_3_^−^, mmol/L	0.03	0.533	0.02	0.631
Net acid Excretion, mEq/24 h	−0.23	<0.001	−0.19	<0.001
Endocrinology				
Thyroxine pmol/L	−0.05	0.239	−0.07	0.086
Triiodothyronine, pmol/L	−0.03	0.476	−0.03	0.533
Growth hormone, ng/mL	0.14	0.001	0.11	0.007
In men	0.20	<0.001	0.21	<0.001
In women	−0.05	0.475	−0.04	0.599

FSR, fractional sulfate reabsorption; RTR, renal transplant recipients; NSAID, nonsteroidal anti‐inflammatory drug.

a Adjusted for potential confounders, including age, gender, smoking, BMI, proteinuria, total energy intake and primary renal disease.

In the association with FSR, significant interaction was found between gender and growth hormone. After splitting on gender, FSR associated positively with growth hormone (*β* = 0.21; *P* < 0.001) in men, while no association was present in women (*β* = −0.04; *P* = 0.599).

## Discussion

To the best of our knowledge, this is the first study that comprehensively assessed plasma sulfate concentrations and sulfate reabsorption in healthy individuals and stable RTR. Compared to kidney donors, plasma sulfate concentrations in RTR were significantly higher, while TSR and FSR were significantly lower. In kidney donors, kidney donation increased plasma sulfate and reduced both TSR and FSR. Furthermore, in both RTR and kidney donors TSR and FSR were found to associate with renal function. In addition, we confirmed some, but not all, of the hormonal and non‐hormonal factors known to affect renal sulfate reabsorption in animal and in vitro studies, as determinants of sulfate reabsorption in RTR.

In humans, most of the inorganic sulfate in the circulation is generated from the sulfur containing amino acids methionine and cysteine, which are derived from dietary protein (Sabry et al. 1965; Grimble 1994; Houterman et al. 1997). Circulating inorganic sulfate concentrations in plasma are primarily regulated by the kidneys, which filter and extensively reabsorb the cleared sulfate to maintain plasma sulfate concentrations between approximately 0.24 and 0.42 mmol/L (Goudsmit et al. 1939; Hierholzer et al. 1960; Cole and Evrovski 1997; Kock et al. 1997). Plasma sulfate concentrations in kidney donors before donation are in line with these studies. Compared to kidney donors before donation, plasma sulfate is higher in RTR, while TSR and FSR are both lower. Higher plasma sulfate values are likely the consequence of decreased kidney function (Hierholzer et al. 1960), while the lower TSR and FSR values may either be attributed to the use of corticosteroids in RTR, which are known to down‐regulate gene expression of the NaS1 transporter (Renfro et al. 1989), or to reduced renal function, as seen in the kidney donors after donation.

In the kidney donors, the plasma sulfate concentrations increased after kidney donation, whereas TSR and FSR decreased by approximately 50% and 25%, respectively. Since sulfate is freely filtered, the increase in plasma sulfate is to be expected after reduction in renal function. The changes in TSR and FSR can be explained by several mechanisms that underlie sulfate reabsorption. The active process of sulfate reabsorption is regulated by various transporters located in the proximal tubuli (Brazy and Dennis 1981; Lucke et al. 1981; Pritchard and Renfro 1983; Low et al. 1984; Schneider et al. 1984; Turner 1984; Bastlein and Burckhardt 1986; Kuo and Aronson 1988; Markovich and Aronson 2007). However, the rate limiting step is thought to be mediated by the apical sodium sulfate co‐transporter (NaS1), which works at a near maximal rate under physiological conditions (Becker et al. 1960; Mudge et al. 1973). In view of this, the circa 50% reduction in TSR can be explained by the circa 50% reduction in total sodium‐sulfate co‐transporters after kidney donation. When comparing the changes in TSR with tubular reabsorption of potassium and sodium it becomes clear that kidney donation has a more profound effect on sulfate reabsorption than on potassium and sodium reabsorption, further supporting the notion that NaS1 worked at near maximal rates under pre‐donation conditions. The relatively smaller reduction in FSR, compared to TSR, indicates that the decrease in the amount of renal sulfate transporters exceeds the decrease in sulfate supply (i.e., filtered sulfate load) to the kidney. The relatively lower reduction in filtered load can likely be attributed to the hyperfiltration of the remaining kidney, since mGFR decreases by around 35% after kidney donation.

Regarding renal function, in RTR and kidney donors both TSR and FSR were positively associated with renal function, which is in line with previous findings in dogs (Berglund and Lotspeich 1956). For sodium is has been long recognized that the tubular sodium reabsorption is increased at increasing GFR and this is termed the glomerulotubular balance (GTB) (Thomson and Blantz 2008). Though the mechanisms of the GTB aren't fully understood for sodium, it is possible a comparable effect takes place for sulfate, which could explain the findings for TSR. This, however, does not explain the positive association of renal function with FSR.

Unfortunately, there are only a few other studies that studied sulfate reabsorption in men. One of these, infused sulfate in a small group of healthy young adults and showed that the maximum tubular reabsorption of sulfate is rapidly exceeded with increasing plasma sulfate levels. Consequently, the amount of filtered sulfate becomes greater than the amount reabsorbed in tubules (Becker et al. 1960). Based on this, sulfate clearance should asymptotically approach GFR at increasing filtered loads, leading to a decreasing sulfate reabsorption coefficients. Therefore, we expected to find an inverse association between sulfate reabsorption and plasma sulfate. However, our data revealed the opposite, as we found a positive association between plasma sulfate and sulfate reabsorption in our kidney donors and RTR. These findings could indicate that, although sulfate reabsorption is quickly saturated within individuals, in persons with higher plasma sulfate concentrations at steady state, renal sulfate reabsorption is increased, perhaps through upregulation of the NaS1 transporter in these individuals. A plausible physiological reason for up‐regulation of sulfate reabsorption could be an increased sulfate demand, possibly due to increased exposure to drugs and endotoxins that are frequently conjugated with sulfate before they are excreted in urine (Levy 1986; Falany 1997). With reduced renal function, retention of uremic toxins and drugs increases (Meijers and Evenepoel 2011; Lekawanvijit 2015), which may increase the sulfate demand for sulfation of these compounds, as this process promotes renal excretion through increased solubility. Though speculative, an increase in sulfate reabsorption to meet the increased sulfate supply is no unknown phenomenon, as it also occurs during pregnancy, where plasma sulfate levels increase more than twofold through upregulation of the NaS1 transporter (Lind 1980; Dawson et al. 2015).

To further identify the determinants of FSR in RTR, we analyzed hormonal and nonhormonal factors that were shown to influence NaS1 gene transcription in animal and in vitro studies. These factors included: dietary sulfate (Sabry et al. 1965; Neiberger 1991; Grimble 1994; Houterman et al. 1997; Markovich et al. 1998; Sagawa et al. 1998b), NSAID use (Sagawa et al. 1998a), acidosis (Frick and Durasin 1986), Vitamin D depletion(Fernandes et al. 1997), hypothyroidism (Sagawa et al. 1999b), and hypokalemia (Markovich et al. 1999), which down‐regulated the NaS1 transporter whereas sulfate depletion, hyperthyroidism (Tenenhouse et al. 1991), growth hormone (Sagawa et al. 1999a) and vitamin D suppletion (Fernandes et al. 1997) up‐regulated the NaS1 transporter. In RTR, we confirmed dietary sulfate intake (methionine, cysteine and sulfate‐rich sources), growth hormone and vitamin D intake as determinants for RSR. In addition, we found an inverse association for thiosulfate excretion, which is known to be a competitive inhibitor for sulfate transport by NaS1. No associations were found for thyroid hormones, serum pH, serum potassium and NSAID use. It should be noted that in the RTR, the pH, potassium and thyroid values were mostly within reference ranges which could explain the discrepancy between our findings and data from the aforementioned studies.

Strengths of this study include the large sample size of this well‐defined and specific patient group. The presence of many demographical and laboratory parameters enables adjustment for many potential confounders. In addition, the information on many dietary and endocrine parameters facilitated a comprehensive approach towards identification of determinants of sulfate reabsorption in RTR. However, several limitations of this study need to be addressed. For most variables, our results were within the reference range, precluding the assessment of states of deficiencies or excess. Furthermore, we did not have information on total direct sulfate intake. For direct sulfate intake, we relied on certain foodstuffs (e.g., bread) that are known to be high in sulfate content. A third limitation involves the term determinants, in this study we spoke about the determinants of sulfate reabsorption, since this study tried to confirm the results seen in animal and in vitro studies. However, since this is an observational study design, we cannot draw conclusions on causality and should technically speak about bidirectional associations. Additionally, since we did not have data regarding the NaS1 transcription, we could not explore the molecular mechanisms underlying the observed associations. Lastly, due to lack of statistical power, we were unable to conduct the determinants analyses in the kidney donors.

In conclusion, RTR have higher plasma sulfate concentrations and lower TSR and FSR than kidney donors. In kidney donors, kidney donation greatly reduced both TSR and FSR and in both RTR and kidney donors, TSR and FSR were associated with renal function. In addition, this study confirms serum growth hormone, thiosulfate excretion, vitamin D intake and dietary sulfate intake as determinants for FSR in RTR. Future research should determine the physiological mechanisms behind renal sulfate handling in humans and the prognostic value of renal sulfate reabsorption in RTR

## Conflict of Interest

None of the authors reported a conflict of interest related to the study.
